# Supplementary material for: Diversity and Molecular Evolution of Odorant Receptor in Hemipteran Insects
Source: Insects. 2022 Feb 21;13(2):214. doi: 10.3390/insects13020214 (PMC8878081; doi:10.3390/insects13020214)

Figure S1: Phylogenetic relationships of the odorant receptor genes from ten species of Hemiptera.

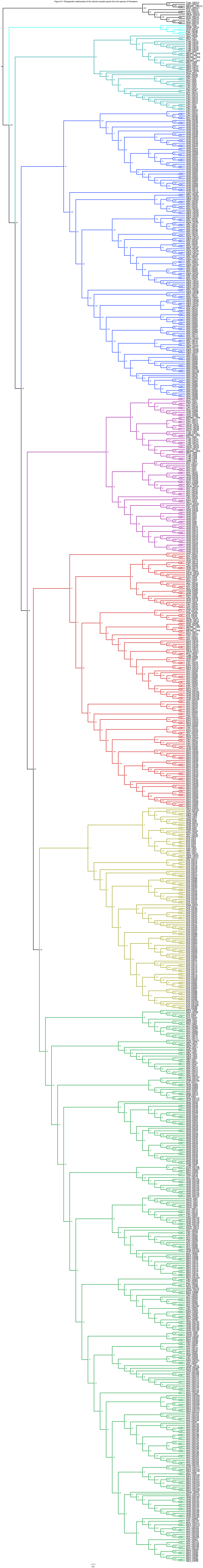

Supplement: Supplementary file 1 [file Figure S1.pdf › Figure S1.pdf]
